# Sleep Deprivation Disrupts Acquisition of Contextual Fear Extinction by Affecting Circadian Oscillation of Hippocampal-Infralimbic proBDNF

**DOI:** 10.1523/ENEURO.0165-19.2019

**Published:** 2019-10-16

**Authors:** Wei Sun, Jia Li, Shuai Cui, Le Luo, Peidong Huang, Chunzhi Tang, Lei An

**Affiliations:** 1 The First Affiliated Hospital of Guizhou University of Traditional Chinese Medicine, Guiyang 550001, China; 2 Medical College of Acupuncture-Moxibustion and Rehabilitation, Guangzhou University of Chinese Medicine, Guangzhou 510006, China; 3 College of Acupuncture-Moxibustion and Orthopedics, Guizhou University of Chinese Medicine, Guiyang 550025, China; 4 Medical College of Acupuncture-Moxibustion and Rehabilitation, Yunnan University of Traditional Chinese Medicine, Kunming 650500, China; 5 Department of Physiology, University of Saskatchewan, Saskatoon, Saskatchewan S7N 5E5, Canada

**Keywords:** circadian oscillations, HPC-IL pathway, neural activity, NMDA receptors, sleep deprivation

## Abstract

Extensive evidence showed that mature brain-derived neurotrophic factor (mBDNF) levels displayed a circadian pattern. Circadian disruption, for example, sleep deprivation (SD), induced functional and behavioral deficits. However, compared with that of mature form, the biological role of the pro-peptide, proBDNF, was poorly understood. Here, we found that proBDNF was expressed under circadian rhythm in the ventral hippocampus (vHPC). SD rats exhibited deficits in acquisition of conditioned extinction and damped rhythmicity in vHPC proBDNF activity that were accompanied by SD between zeitgeber time (ZT)0 and ZT4, but not the late stage of sleep period. Furthermore, SD affected fear extinction through vHPC-IL proBDNF signaling, which was associated with NR2B subunits of NMDA receptors. More importantly, infusion of proBDNF could mitigate SD-induced abnormal neural activity, by suppressing the enhanced basal firing rate of IL-RS and elevating the depressed neural response that evoked by acquisition of conditioned extinction. Therefore, this finding provided the first evidence that circadian oscillation of vHPC proBDNF activity contributed to the effects of SD on acquisition of conditioned fear extinction, and suggested a new therapeutic target to reverse the cognitive deficits in sleep-related mental disorder, such as post-traumatic stress disorder (PTSD).

## Significance Statement

The aim of this study was to assess the circadian role of pro-brain-derived neurotrophic factor (proBDNF) in the ventral hippocampus (vHPC) on contextual fear extinction and detect whether sleep deprivation (SD) affected the circadian rhythm of proBDNF levels and attempted to explore the underlying mechanisms of functional and behavioral deficits. These findings provided the first evidence that circadian oscillation of vHPC proBDNF activity contributed to the effects of SD on acquisition of conditioned fear extinction, and offered potential avenues to mitigate sleep-related cognitive and functional disorders, such as PTSD and depression.

## Introduction

The neurotrophin brain-derived neurotrophic factor (BDNF) is a member of a family of neurotrophic factors critically involved in various physiologic functions, particularly neuroplasticity, memory and sleep (Nakai et al., 2014; Schmitt et al., 2016), suggesting its biological role throughout life (Lu et al., 2009). BDNF protein is biosynthesized as a precursor of BDNF (proBDNF), which can be secreted and processed extracellularly by plasmin or by matrix metalloproteases to produce mature BDNF (mBDNF; Gray and Ellis, 2008). Recent studies in rodents indicated the endogenous cyclical alterations in central mBDNF, its cognate receptor, and other related candidate effectors during sleep were necessary for learning and memory consolidation (Eckel-Mahan et al., 2008; Jang et al., 2010; Zhang et al., 2013). As the pro-peptide of mBDNF, it is quite possible that proBDNF plays a critical role in the regulation of circadian rhythms.

Circadian rhythms were seen in the sleep and wake cycle as well as in several other aspects of physiologic functioning, such as strengthening and integration of newly formed memories (Eckel-Mahan et al., 2008; Gerstner and Yin, 2010; Michel et al., 2013), including fear memories (Pace-Schott et al., 2015; Boyce et al., 2016). According to the synaptic-homeostasis hypothesis, homeostatic regulation of synapse formation during sleep was attributed to reactivation of the underlying neuronal memory traces (Griffith and Rosbash, 2008; Vyazovskiy et al., 2008). Indeed, wakefulness appeared to be associated with synaptic potentiation, whereas sleep may favor global synaptic depression, thereby preserving an overall balance of synaptic strength (Vyazovskiy et al., 2008). This view was also supported by the prominent neuronal feature of sleep in acquainting new information during the following wake-phase (Marshall et al., 2014; Li et al., 2017).

Accumulating evidence has shown that proBDNF and mBDNF elicited seemingly opposite biological effect on modulating brain structure and function (Holm et al., 2009; Je et al., 2012; Mizui et al., 2015). Recently, there was growing interest on the mechanisms of neurotrophin effects on plasticity underlying fear extinction learning (Cowansage et al., 2010; Andero and Ressler, 2012). Distinct from the enhanced effect of mBDNF on LTP, proBDNF can negatively regulate neural remodeling by facilitating the induction of LTD (Yang et al., 2014), which weaken synaptic transmission to enhance learning (Dalton et al., 2012; Walker et al., 2015) and memory (Dalton et al., 2012; Li et al., 2016) of fear extinction. Considering that the critical role of sleep in extinction memory was associated with BDNF signaling (Hill et al., 2016) and the negative retrograde effect of proBDNF on dendritic complexity and spine density (Yang et al., 2014), synaptic transmission (Yang et al., 2009), and cell survival (Je et al., 2012; Sun et al., 2012) was strongly linked to memory decay, it remained unclear whether sleep-dependent circadian rhythms of proBDNF expression played a pivotal role in fear extinction.

Empirical researches have shown that the amygdala, hippocampus, and mPFC were involved in fear conditioning (Sotres-Bayon et al., 2009; Milad and Quirk, 2012). As one of important BDNF-containing inputs, ventral hippocampus (vHPC) was important for supplying BDNF to the IL mPFC to facilitate context-dependent fear learning (Wiltgen et al., 2006; Peters et al., 2010) and extinction (Corcoran et al., 2005; Sierra-Mercado et al., 2011). At the cellular level, increased excitability in infralimbic cortex (IL-PFC) following extinction has been shown to predict extinction success (Burgos-Robles et al., 2007; Santini et al., 2008). Hippocampal mBDNF infusion increased IL-PFC neuron ﬁring (Rosas-Vidal et al., 2014), suggesting that coordinated mBDNF release in the HPC-mPFC circuit. Therefore, the dysfunction of homeostatic regulation induced by sleep pressure may change the cortical firing correlate of behavioral states. Also unknown is which proBDNF-mediated pathway was mainly correlated with SD-induced fear extinction acquisition?

To address above issues, we detected the circadian variations of proBDNF in the vHPC and the effect of SD on it. By pharmacological infusions, we attempted to verified fear extinction learning was dependent on the intact circadian of vHPC proBDNF expression. Considering the molecular mechanism linking BDNF/TrkB signaling with NMDA receptors in memory (Nakai et al., 2014; Itoh et al., 2016), we also infused antagonist of NMDA-2A or NMDA-2B subunit into vHPC during SD to detect the interaction between proBDNF and NMDA receptors during fear extinction. Finally, by single-unit recording, we inspected that the circadian oscillation of proBDNF level was modified within HPC [or basolateral amygdala (BLA)]-IL circuit. Our findings supported the hypothesis that the circadian oscillation of proBDNF activity in vHPC was critical for fear extinction acquisition, while the interference in the circadian peak of proBDNF expression by SD pointed a core feature of the mental disorder.

## Materials and Methods

### Animals and drug administration

Three-month-old male Sprague Dawley rats (Beijing Research Center for Experimental Animals) were individually housed in standard Macrolon cages with free access to food and water unless food was restricted to prepare for level press experiment. Animals were kept on a 12/12 h light/dark cycle (L/D; lights on at 8 A.M.) unless otherwise specified. A layer of sawdust served as bedding. During the light phase, stalls were lit by two 200-W ﬂuorescent light bulbs that produced an average light intensity of 300–400 lux at the level of cages. Lux was detected using a light meter (VWR Scientific). Each animal was handled extensively (10 min/d) till the experimental day. All experiments were performed according to the ethics Committee on the Care and were approved by the University of Animal Research Ethics Board.

Groups and treatments were indicated in figure and its legend. All control animals were treated with the same dose of saline or artificial CSF (ACSF).

### Sleep deprivation (SD)

Three to 7 d before being used, all animals were transferred to a testing room controlled for temperature (21–23°C), relative humidity (40–50%), and noise (40–50 dBA during the light phase). Subgroups of rats were regularly monitored for their sleep-wake behavior via the infrared detector (Mini Mitter) was attached to each cage to detect activity. Infrared beam breakage was recorded using VitalView Data Acquisition System (Mini Mitter). Data were transformed into bins per min using ClockLab (Actimetrics). The actograms shown were plotted the locomotion of rats in the corresponding L/D or D/D conditions. The locomotor activity during light or dark phase was measured. All data were normalized by the activity of control group during the light phase.

Rats were subjected to 4-h SD from either zeitgeber time (ZT)0 to ZT4 or ZT4 to ZT8. These time window were chosen dependent on circadian oscillation of proBDNF activity during the light phase (see Results) and the previous findings in sleep’s role in learning and memory (Carter et al., 2009; Yang et al., 2012; Ackermann and Rasch, 2014; Ravassard et al., 2016; Vyazovskiy et al., 2017). For example, early studies indicated that the early period of sleep (such as from ZT0 to ZT4; Carter et al., 2009; Vyazovskiy et al., 2017) was critical for promotion of naturally acquired extinction (Rasch et al., 2007; Yang et al., 2012; Pace-Schott et al., 2015), erasing fear information (Rolls et al., 2013; Ravassard et al., 2016), and enhancing new learning (Rasch and Born, 2013; Stickgold and Walker, 2013; Landmann et al., 2014). However, the second half of the night was important for mirror tracing, priming, and implicit memory (Rasch and Born, 2013; Ackermann and Rasch, 2014). Briefly, animals were kept under constant observation of the experimenter and kept awake by mild sensory stimulation, which involved tapping on the cage, gently shaking the cage or, if necessary, disturbing the sleeping nest, when they assumed a sleeping posture (Hagewoud et al., 2010; Oyanedel et al., 2015). Previous studies have shown that this procedure was effective in keeping rodents awake for several hours, as established by electroencephalic recordings (Oyanedel et al., 2015), without being a major stressor (Tadavarty et al., 2009). The number of stimuli needed to keep the rats awake was recorded.

### Fear conditioning

Rats were contextual fear conditioned and extinguished in standard operant chambers (Coulbourn Instruments) inside sound-attenuating boxes (Med Associates) in an isolated behavioral room. The floor of the chambers consisted of stainless-steel bars that delivered a scrambled electric footshock. Between experiments, shock grids and floor trays were cleaned with soap and water, and chamber walls were cleaned with wet paper towels. On each of three consecutive acclimation days, rats were placed into the test chambers for 10 min and then returned to their home cages. For fear conditioning, rats received five habituation tones (4 kHz, 30 s, 77 dB), immediately followed by six conditioning tones that co-terminated with footshocks (0.5 s, 0.5 mA). The intertrial interval varied around 3 min and showed in blocks of two trials. Extinction training was conducted in the same context used for conditioning and began either 48 h (light phase), 60 h (dark phase), or 72 h (light phase) post-conditioning. Extinction training was conducted in the same context used for conditioning training while no shock was trigged during extinction training. All data were shown in intertrial interval blocks.

Fear behavior was assessed offline from videos by measuring freezing with the exception of respiratory movements, which was an innate defensive behavior (Blanchard and Blanchard, 1969). One unit of freezing was defined as a continuous absence of movement in 1 s sampled every 5 s. The value was expressed as the percentage of the total number of observations.

### Locomotion and anxiety test

A subset of rats tested in the fear conditioning experiment was assessed locomotor activity in a 20-min open field session within an isolated testing room as described previously (Mueller et al., 2010; Peters et al., 2010). The open field consisted of a 91.5 × 91.5 × 61 cm Perspex box with dark walls and a white floor was dimly illuminated. Grid lines divided the open field into a peripheral region (within 15.25 cm of the walls) and central region (61 × 61 cm) of approximately equal area. Rats were released from the middle of the open field. The distance traveled and the time spent within peripheral/central region were recorded using VersaMax Activity Monitoring System (AccuScan Instruments).

### Motivation test

On completion of fear conditioning test, a subset of rats was food restricted at 85% of free-feeding weight. Rats were trained to lever press for 45-mg food pellets in standard operant chambers located inside sound-attenuating boxes (Med Associates). The chambers contained two retractable levers located on either side of a central food trough. White cue lights were located above each lever. Delivery of food pellets was accompanied by an auditory stimulus and illumination of a cue light located within the food trough. As previous studies (Paterson et al., 2005; Peters et al., 2010), animals initially received 45-mg food pellets at a 30-s interval for 30-min initial sessions with no requirement to lever press. When rats consumed all pellets during initial stage, they were trained daily 30-min (between ZT3.5 and ZT4.5) sessions with one of two levers extended randomly when the cue light above the level was illuminated. The schedule was progressively changed according to the sequence fixed ratio (FR)-1, FR-15, FR-30, and finally FR-60. The criterion for moving through the sequence was earning 50 pellets within the 30-min session. Rats were tested in a 30-min session till they reached 10 presses per minute on FR-60.

### Bilateral microinjection

Surgery was prepared as our reports (An and Sun, 2017, 2018). Briefly, rats were anesthetized with sodium pentobarbital (60 mg/kg, i.p.), placed in a stereotaxic frame (SN-3, Narishige) for surgery under atropine (0.1 mg/kg, i.p.) which help relieve respiratory congestion. Stainless-steel guide cannulae (22 gauge; Plastics One, Inc.) were bilaterally inserted above two of three sites: vHPC (AP: –6.0 mm, ML: ±5.0 mm, DV: 5.4–5.6 mm), BLA (AP: –2.8 mm, ML: ±5.0 mm, DV: 7.4–7.6 mm), and IL-PFC (AP: +2.8 mm, ML: ±3.1 mm, DV: 3.8–4.2 mm; angled at 30°). For IL cannula implantation, the angled approach was used to avoid backflow to PL (Sierra-Mercado et al., 2011). Sterile stainless-steel stylet (30 gauge, 10 mm, Plastics One Inc.) was inserted into guide cannula to avoid obstruction. All rats were allowed to recover for at least 7 d.

Infusions were achieved by inserting 30-gauge dummy needles (10 mm, Small Parts Inc.) linked via PE-50 tubing to a microsyringe pump (Harvard Apparatus), extended 1.0 mm beyond the end of the cannulae. Needles were inserted into both cannula then infused cleavage-resistant proBDNF (2 ng/ml), sheep anti-proBDNF antibody (10 μg/μl), mBDNF [1.5 µg/µl; 3-(2-carboxypiperazin-4-yl)propyl-1-phosphonic acid (CPP; 32 ng/μl)], NVP-AAM077 (0.8 ng/μl), Ro25-6981 (2.0 ng/μl), or ACSF into vHPC, BLA, or IL-PFC area was initiated at 0.5 μl/min per side for 2 min. The drugs were purchased from Sigma-Aldrich Chemicals except for cleavage-resistant proBDNF (Alomone Labs), anti-proBDNF antibody (R&D Systems), and human mBDNF (R&D Systems). All doses were determined from published studies, which indicated the safety and efficacy of cleavage-resistant proBDNF (Woo et al., 2005; Holm et al., 2009), anti-proBDNF antibody (Fan et al., 2008; Bai et al., 2016), mBDNF (Peters et al., 2010; Rosas-Vidal et al., 2014), CPP (McDonald et al., 2005; Dong et al., 2013), NVP-AAM077 (Fox et al., 2006; Dong et al., 2013), and Ro25-6981 (Fox et al., 2006; Dong et al., 2013). The infusion needles were left in place for a 5-min period to permit diffusion of the drug. One week before commencement of the experiment, habituation sessions were conducted four times for each rat without infusion.

Following testing in all conditions, the rats were sacrificed with urethane and the brain was collected in 10% formalin/10% sucrose. The placements of cannulae were used to identify the location of the infusion sites. Only data obtained from rats with correctly inserted needles were included in statistical analysis.

### Protein preparations and Western blot analysis

Additional groups of rats were housed in a L/D for at least two weeks before time course experiment. Rats were killed every 4 h during one 24-h period. During the dark cycle, rats were killed under a dim red light (1–2 lux). vHPC, BLA, and IL-PFC were rapidly dissected on ice and snap-frozen in liquid nitrogen and stored at –80°C. Tissues was homogenized in ice-cold lysis buffer (pH 7.4) containing a cocktail of protein phosphatase and proteinase inhibitors (Sigma) to avoid dephosphorylation and degradation of proteins. Protein concentrations were detected using the bicinchoninic acid (BCA) assay. Twenty micrograms (15 μl) of total protein per lane was resolved in 10–15% SDS-PAGE gels followed by electro-transferring to PVDF membranes (Pall). Non-specific binding of antibodies to membranes was blocked with 5% (w/v) non-fat milk for 2 h at room temperature, followed by incubation overnight at 4°C with the primary mouse anti-proBDNF antibody (1:500; Santa Cruz Biotechnology) or rabbit anti-mBDNF antibody (1:5000; Millipore Bioscience Research Reagents). Mouse anti-β-actin (1:20,000; Sigma) was used as an internal control. After rinsing in Tris-buffered saline-Tween 20, the membranes were incubated with horseradish-peroxidase (HRP)-conjugated secondary goat anti-mouse or anti-rabbit IgG (1:1000; Southern Biotechnology Associates) incubated for 2 h at room temperature. After three rinses (10 min in each) in TBS-T buffer, immunoreactivity was detected by ECL Western Blotting Detection kit (CWBIO).

### Immunohistochemical staining

Rats were anesthetized and transcardially perfused with saline, then ﬁxed with 4% paraformaldehyde in PBS. The intact brains were post-ﬁxed in 4% paraformaldehyde for an additional 2 h, and placed in 30% sucrose cryoprotectant solution for 48 h. Then the brains were embedded in OCT medium and stored at 80°C. Sagittal sections (10 μm thick) were prepared using a Leica microtome (Leica RM2235, Leica Biosystems). In brief, deparaffinized sections were rehydrated through a graded series of ethanol to PBS. Antigen retrieval was performed in citrate buffer (10 mM, pH 6.0) at 99°C for 40 min. Sections were incubated in 3% H_2_O_2_ to clear endogenous peroxidase, blocked and incubated overnight with chicken anti-proBDNF antibody (1:000, Millipore Bioscience Research Reagents) at 4°C. Negative controls were conducted by exchange of primary antibody for PBS. Biotinylated-conjugated anti-chicken IgG (1:1000; Millipore Bioscience Research Reagents) was incubated for 1 h at 37°C and then visualized with DAB (Sigma-Aldrich). Between steps, the sections were washed twice in PBS. Finally, the sections were counterstained with hematoxylin and mounted. Sections were viewed on a Leica microscope and digitized using a charge-coupled device camera (Olympus DP71). Quantiﬁcation was performed using Image-Pro Plus software. One section of each region was selected from each rat in each group, and ﬁve ﬁelds per section were taken for analysis. We examined the sum of integrated optical density (IOD) of proBDNF-positive neurons in each ﬁeld of a selected section.

### Single-unit recording

Microelectrode array was arranged in a 4 × 8 matrix using 25-μm-diameter tungsten wires (California Fine Wires) in a 35-gauge silica tube (World Precision Instruments). It was then attached via gold pins to an EIB-36-PTB board (Neuralynx Inc.). The electrode tips were gold-plated to maintain the impedance to 200–600 kΩ measured at 1 kHz.

Rats were anesthetized with isoflurane and prepared for surgery using previously reported procedures (An and Sun, 2018; Sun et al., 2018a). Cannula were implanted bilaterally into vHPC (AP: –6.0 mm, ML: ±5.0 mm, DV: 5.4–5.6 mm). Electrode arrays were unilaterally implanted into IL-PFC (AP: +3.0 mm, ML: ±0.6 mm, DV: 4.5–5.0). The hemisphere was implanted randomly but counterbalanced across rats of groups. A stainless-steel wire was used as ground electrode and soldered onto one jewelers' screw, which was implanted into the skull. The electrode array was fastened to the cranium by dental acrylic with additional scull screws as anchors.

During the whole test, nine rats were assigned to one of three conditions (e.g., SD, CON, SD-proBDNF) in each recording session. This was assigned in a pseudorandom order between subjects and treatments during each training day. The recording was conducted for 5 min in the fear conditioning chamber immediately following the extinction training with a Digital Cheetah system (Cheetah software, Neuralynx Inc.). Additionally, a 30-min baseline recording was performed in the fear conditioning chamber 30 min before fear extinction training. Unit signals were recorded via a HS-36 unit gain headstage (Neuralynx Inc.) mounted on animal’s head by means of lightweight cabling. Unit activity was amplified (1000–10,000 times), sampled at 32 kHz and 600- to 6000-Hz bandpass filters. To verify the stability of recording, unit activities were recorded for ∼15 min before the training initiation.

After the completion of all recording sessions, electrolytic lesions (10-μA current for 10 s) were made to mark the selective recording sites, which were identified using standard protocols with reference to The Rat Brain in Stereotaxic Coordinates (Paxinos and Watson, 1997). Only data from rats with probes contained within IL-PFC were analyzed.

### Unit isolation and classification criteria

Spike sorting was performed with offline Neuralynx’s software (SpikeSort 3D), using a combination of KlustaKwik, followed by manual adjustment of the clusters (Klusters software package). Briefly, multiple parameters were used to effectively visualize clusters with the most often used combination of spike height, trough, and energy, associated with the waveforms (Insel and Barnes, 2015; Kim et al., 2016). Each cluster was then checked manually to ensure that the cluster boundaries were well separated and wave form shapes were consistent with action potentials (Fig. 6*C*). Interspike interval histograms were additionally examined for ensuring single unit activity (Fig. 6*B*). Using methods described elsewhere (Insel and Barnes, 2015; Kim et al., 2016; An et al., 2018; Sun et al., 2018b), units were then graded for quality and classiﬁed as regular-spiking projection neuron (RS) and fast-spiking inhibitory neuron (FS) from PFC recording, respectively.

### Code accessibility

All code is available as extended data. Code for data processing and analysis can be found at http://neurosuite.sourceforge.net/. We used SpikeSort 3D, Neuroexplorer and MATLAB software for data processing. These were run in Windows 7 OS. All Data are available from the corresponding author upon reasonable request.

10.1523/ENEURO.0165-19.2019.ed1 Extended Data Supplementary The codes. Download Extended Data 1, ZIP file.

### Statistical analysis

Data are expressed as mean ± standard error of the mean. The analyses were performed with Neuroexplorer (Nex Technologies), MATLAB (MathWorks), and SPSS 17.0 software. The data of fear conditioning test were compared using a repeated-measures ANOVA as a two-way design in which testing block (block 1, block 2, block 3, block 4, block 5, or block 6) was a within subjects measure, and treatment (control, SD, or other treatments) was a between subjects measure. A one-way ANOVA examined the effects of treatment (control or SD) on the data of open field and motivation tests. The data of Western blot and immunohistochemical tests were subjected to a two-way ANOVA in which brain region (IL-PFC, BLA, or other brain areas) and treatment (control, SD, or other treatments) were dependent variables. To measure the correlation between average firing rate and the ratio of the spike wave form, Pearson’s correlation analysis was performed on unit classification. For comparison of the mean firing rate in the electrophysiological recording, a repeated-measures ANOVA was employed to examine the effects of testing block (block 1, block 2, block 3, block 4, or block 5) as a within subjects measure, and the effects of treatment (control, SD, or SD plus proBDNF treatment) as a between subjects measure. When the ANOVA revealed a signiﬁcant main effect or interaction between main factors, data were further analyzed by Tukey’s *post hoc* tests; *p* < 0.05 level, *p* < 0.01 level, and *p* < 0.001 level of confidence were used in the analyses.

## Results

### SD inhibited proBDNF activity rise during circadian cycle

**Figure 1.**
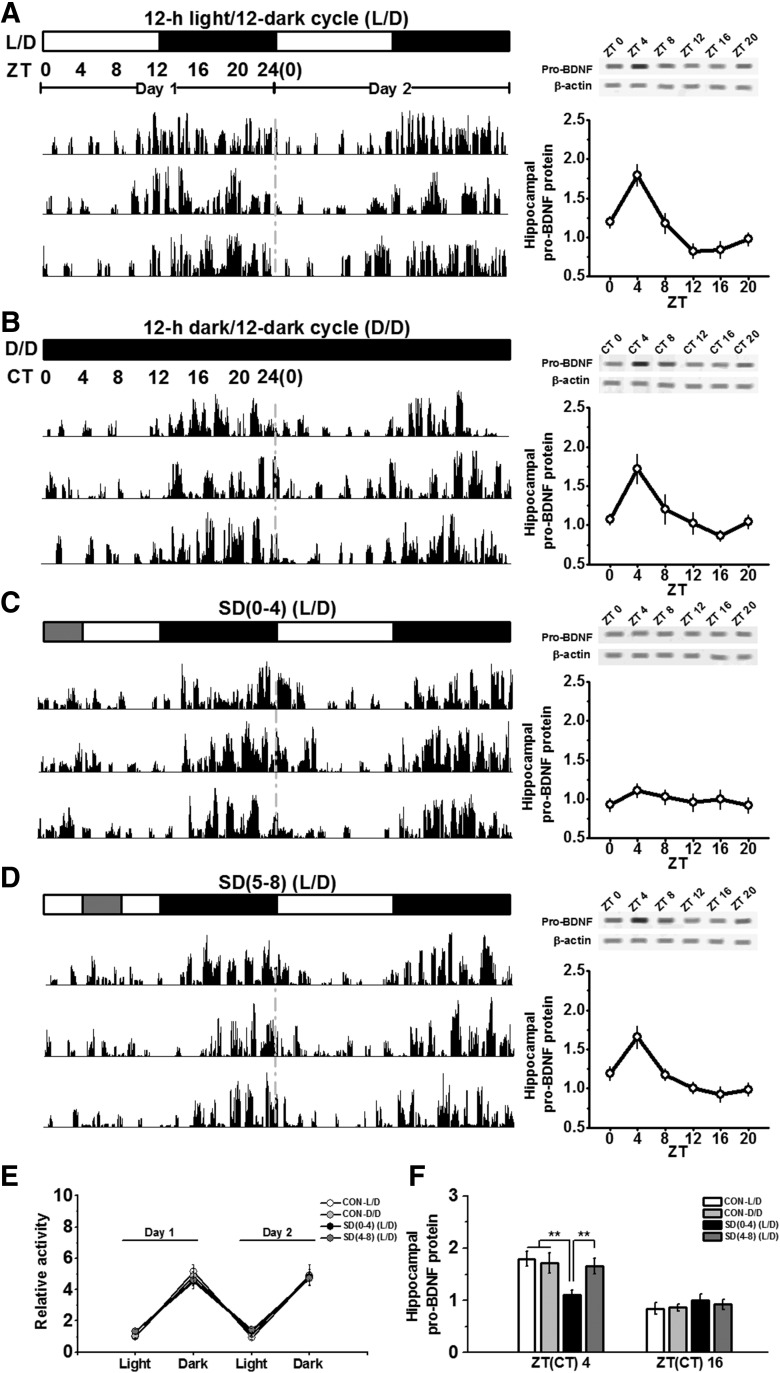
SD interrupted circadian oscillations in proBDNF activity in the hippocampus. Rats were entrained to a standard L/D condition (***A***), 12/12 h dark/dark cycle (D/D condition; ***B***), partial SD between ZT0 and ZT4 [SD(0–4); ***C***], or partial SD between ZT4 and ZT8 [SD(4–8); ***D***]. Actograms of voluntary movement during L/D (free-running) condition (***A***, left), D/D (free-running) condition (***B***, left), SD(0–4) condition and SD(4–8) condition (***D***, left) showed a circadian oscillation. Each actogram was from individual animal; *n* = 6–8 per group. The expression of vHPC proBDNF, normalized to actin, during the 24-h cycle in L/D (free-running) condition (***A***, right), D/D (free-running) condition (***B***, right), SD(0–4) condition (***C***, right), and SD(4–8) condition (***D***, right); *n* = 7–8 per time point. ***E***, When ZT were blocked into 12-h increments, the movement, normalized by the activity of L/D condition during ZT0 and ZT12, showed no different among all conditions; *n* = 6–8 per group. ***F***, Quantiﬁcation of proBDNF relative to actin protein expression at ZT4 and ZT16; **p < 0.01; *n* = 7–8 per time point.

To determine whether rats entrained to a L/D have oscillations in hippocampal proBDNF activity, vHPC tissues were taken every 4 h starting at light onset (ZT0). During the dark cycle, rats were killed under red light to avoid the influence of light-induced activation on neurotrophin signaling (Chang et al., 2014). As shown in Figure 1, actograms generated from monitoring voluntary activity of rats in L/D condition (Fig. 1*A*, left) indicated that locomotion rhythms of L/D rats maintained a circadian oscillation (Fig. 1*E*). Western blot analysis showed a pronounced oscillation in proBDNF expression over the 24-h period (Fig. 1*A*, right). Since the expression of proBDNF was signiﬁcantly higher at ZT4 compared with ZT16 (*p* < 0.001), ZT4 and ZT16 were used as time points for subsequent comparison (Fig. 1*F*). To verify that the L/D oscillations in proBDNF activity were truly circadian, we tested whether or not they persisted under free-running (D/D) condition (Fig. 1*B*). Mice were entrained to an L/D cycle for two weeks and then placed in complete darkness (D/D) for at least two weeks before being analyzed. Similar actograms were observed (Fig. 1*B*, left), locomotor activity of L/D rats was comparable to that of D/D rats (Fig. 1*E*). As expected, in the D/D condition, vHPC proBDNF at circadian times (CT) 0, 4, 8, 12, 16 and 20 maintained circadian variations (Fig. 1*B*, right), which were in a similar pattern as the L/D rats’ (Fig. 1*F*). No sign of circadian rhythmicity in proBDNF activity exhibited in striatal or cerebellar tissue (data not shown), indicating either that there was no proBDNF oscillation in striatal or cerebellar neurons, or that there was no synchronized activity in these regions.

SD was conducted on rats between ZT0 and ZT4 [SD(0–4)] to seek whether SD could perturb the circadian variations in pro BDNF activity (Fig. 1*C*). Although the locomotion activity of SD(0–4) rats during the SD period was slightly higher than rats in the L/D and D/D condition, no statistical difference was found (Fig. 1*E*). Strikingly, proBDNF activity of SD(0–4) rats lost marked 24-h variations in the L/D condition of the day following SD (Fig. 1*C*, right) and the expression at ZT4 was significantly lower than other groups (all comparisons: *p* < 0.01; Fig. 1*F*). To determine the specific time effect of SD on circadian oscillations in proBDNF activity, we performed SD between ZT4 and ZT8 [SD(4–8)], which was the decaying period in circadian variations of proBDNF activity. However, SD from ZT4 to ZT8 did not affect rats’ voluntary movement during L/D condition (Fig. 1*D*, left) and the synchronized oscillations in proBDNF expression (Fig. 1*D*, right, F) on the following day. Additionally, there was no difference between SD(0–4) and SD(4–8) conditions in the number of stimuli to keep the rats awake (data not shown), suggesting that the stimulant effects of tapping or shaking were not attributed to the circadian variation .

### SD affected extinction of conditioned fear, but not acquisition, of fear conditioning

**Figure 2.**
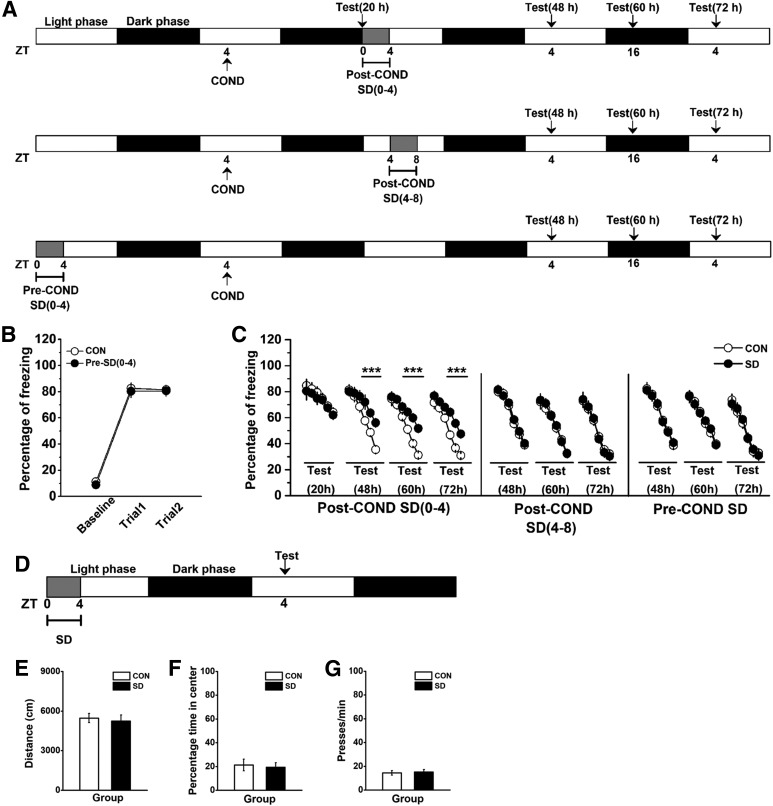
SD between ZT0 and ZT4 disturbed acquisition of extinction but not locomotion, anxiety or motivation behavior. ***A***, Treatment groups and procedure. Contextual fear conditioning was performed 1 d before SD, which was conducted between ZT0 and ZT4 (top). Contextual fear conditioning was performed 1 d before SD, which was conducted between ZT4 and ZT8 (middle). SD, which was performed between ZT0 and ZT4, was conducted 1 d before fear conditioning (bottom). Test and time signs indicated the time-points for fear extinction training following acquisition of fear conditioning. ***B***, A similar effect was observed between pre-SD(0–4) and control groups in freezing levels during acquisition of fear conditioning; *n* = 12 for each group. ***C***, Fear extinction learning between SD and CON groups in Post-COND SD(0–4) and SD(4–8), and Pre-COND SD conditions; ***p < 0.001; *n* = 5–8 per test. ***D***, Rats were SD between ZT0 and ZT4, and tested at ZT4 on the next day. Locomotion was defined as total travel distance in an open field, and anxiety was defined as percentage time in the center of the apparatus. For motivation, rats were placed in the testing chamber and allowed to press for food on a FR-60 schedule of reinforcement. SD did not significant affect travel distance (***E***), the percentage time spent in the center of the apparatus (***F***), and press time per min in the test chamber (***G***); *n* = 8 per group.

We also tested whether the effect of SD on proBDNF oscillations affected fear conditioning, including extinction [post-COND-SD(0–4) (Fig. 2*A*, top); post-COND-SD(4–8) (Fig. 2*A*, middle)] and acquisition [pre-COND-SD(0–4); Fig. 2*A*, bottom]. Rats trained for contextual fear were sleep deprived between ZT0 and ZT4 on the following day, and then tested extinction of conditioned fear 20 h (pre-extinction at ZT24), 48 h (light phase at ZT4), 60 h (dark phase at ZT16), or 72 h (light phase at ZT4) post-training. No group difference was observed when fear extinction training was conducted before SD [effect of treatment: *F*_(1,8)_ = 1.56; *p* > 0.05; the first subset of post-COND-SD(0–4) in Fig. 2*C*]. Similarly, there was no statistical difference in the freezing levels between CON and SD groups in the Post-COND SD(4–8) or Pre-COND condition (data not shown). However, SD(0–4) rats tested during both light [ZT4; effect of treatment: *F*_(1,14)_ = 29.72, *p* < 0.001; block 3–5: *p* < 0.001; the second subset of post-COND-SD(0–4) in Fig. 2*C*] and dark (ZT16) phases [effect of treatment: *F*_(1,12)_ = 43.10, *p* < 0.001; block 3–5: *p* < 0.001; the third subset of post-COND-SD(0–4) in Fig. 2*C*] had deﬁcits in extinction training compared with their matched control rats. This effect can be persistently tested on the third day following conditioning training [72 h, effect of treatment: *F*_(1,10)_ = 38.86, *p* < 0.001; block 3–5: *post hoc p* < 0.001; the last subset of post-COND-SD(0–4) in Fig. 2*C*], predicting the persistent effect of SD on extinction acquisition. Furthermore, SD during ZT4 and ZT8 following conditioning training did not damage acquisition of fear extinction, which tested 48 h [effect of treatment: *F*_(1,12)_ = 1.12, *p* > 0.05; the subset of post-COND-SD(4–8) in Fig. 2*C*], 60 h (effect of treatment: *F*_(1,12)_ = 1.33, *p* > 0.05) or 72 h (effect of treatment: *F*_(1,12)_ = 1.17, *p* > 0.05) after conditioning training. When SD was conducted 24 h before the acquisition of fear conditioning, no difference was found during acquisition (effect of treatment: *F*_(1,22)_ = 0.83, *p* > 0.05; Fig. 2*B*) or extinction training that tested at 48 h (ZT4; effect of treatment: *F*_(1,10)_ = 0.94, *p* > 0.05; the last subsets of pre-COND-SD in Fig. 2*C*), 60 h (ZT16; effect of treatment: *F*_(1,10)_ = 1.29, *p* > 0.05) or 72 h (ZT4; effect of treatment: *F*_(1,10)_ = 1.31, *p* > 0.05). Additionally, the effects of SD on locomotion, anxiety, and motivation behavior were tested 24 h after SD (Fig. 2*D*). No statistical difference was found in the total travel distance (Fig. 2*E*) and the percentage time in the center of the apparatus (Fig. 2*F*) in the open field test, or the motivation behavior (Fig. 2*G*). Therefore, the alternation in freezing behavior was not attributed to the effect of SD on locomotion, anxiety or motivation defects.

**Figure 3.**
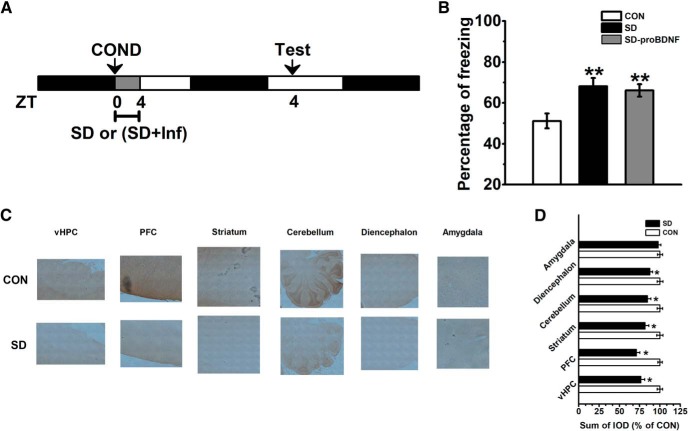
SD-induced consolidation memory deficit was not attributed to its effect on circadian proBDNF expression. ***A***, Conditioned rats were subjected to 4-h SD with or without proBDNF infusion ZT0. At the ZT4 of the following day, to tested fear memory, rats were returned to the same contextual chambers and presented with as few as one tone. ***B***, SD impaired memory consolidation of conditioned fear, while injection of cleavage-resistant proBDNF into the vHPC did not rescue the defect; **p < 0.01 versus CON; *n* = 7 per group. Immunohistochemical detection of proBDNF in vHPC, PFC, striatum, cerebellum, diencephalon and amygdala of SD and control rats. Digital light micrographs (20×) of proBDNF-positive cells (***C***) and sum of IOD comparison (***D***); *p < 0.05 versus CON; *n* = 5 per group.

Furthermore, rats were SD between ZT0 and ZT4 immediately following conditioning training (Fig. 3*A*). We found SD severely disturbed memory consolidation of conditioned fear (effect of treatment: *F*_(2,18)_ = 24.58, *p* < 0.001; *post hoc p* < 0.01; Fig. 3*B*). This deficit cannot be reversed by the infusion of proBDNF (*post hoc p* < 0.01; Fig. 3*B*). The immunohistochemical staining with anti-proBDNF antibody was conducted 24 h after extinction training. Compared with SD group, the expression of proBDNF was significantly elevated in the brain regions of control group (Fig. 3*C*), including vHPC, PFC, striatum, cerebellum, and diencephalon (for all comparisons *p* < 0.05; Fig. 3*D*), which indicated that proBDNF acts at different brain areas during the consolidation process. However, no statistical difference was found in the amygdala. Meanwhile, a positive correlation between the level of proBDNF in the vHPC and fear behavior was found (Pearson’s *r* = 0.216; *p* < 0.001). Together with the finding of circadian variations of proBDNF activity, our results implied the correlation between the oscillations of proBDNF activity in the vHPC and fear extinction, but not initial formation or consolidation fear memory.

### SD impaired circadian proBDNF level through NR2B-containing NMDA receptors

**Figure 4.**
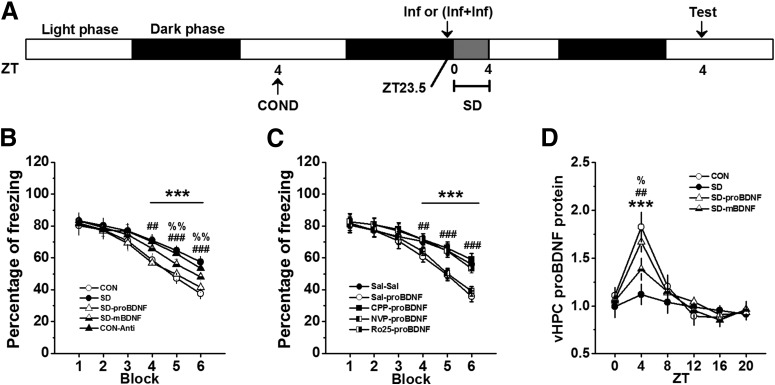
The mitigating effect of proBDNF on SD-induced deficits in acquisition of fear extinction required NR2B subunit of NMDA receptors. ***A***, Treatment procedure. Conditioned rats received post-SD infusion(s) and then tested 48 h following contextual conditioning. ***B***, The effect of proBDNF, mBDNF, and anti-proBDNF antibody on SD-induced extinction impairment; ***p < 0.001: SD or CON-Anti versus CON or SD-proBDNF; ##*p* < 0.01, ###*p* < 0.001: SD-mBDNF versus CON or SD-proBDNF; %%*p* < 0.01: SD-mBDNF versus SD or CON-Anti; *n* = 7 per group. ***C***, Rats were subjected SD between ZT0 and ZT4, the effect of co-infusion NMDA receptor antigonists (CPP, NVP, and Ro25) and proBDNF on freezing levels were tested; ***p < 0.001: Sal-proBDNF versus Sal-Sal, CPP-proBDNF or Ro25-proBDNF; ##*p* < 0.01, ###*p* < 0.001: NVP-proBDNF versus Sal-Sal, CPP-proBDNF or Ro25-proBDNF; *n* = 8 per group. ***D***, The proBDNF levels of SD rats that treated with proBDNF and mBDNF throughout the circadian cycle; ***p < 0.001: SD versus CON or SD-proBDNF; ##*p* < 0.01: SD-mBDNF versus CON or SD-proBDNF; %*p* < 0.05: SD-mBDNF versus SD; *n* = 6 per time point.

To verify the effect of the circadian proBDNF level on fear extinction and whether exogenous proBDNF could mitigate extinction deficits induced by SD, rats were subjected to contextual fear conditioning and, the following day, received bilateral vHPC infusion of cleavage-resistant proBDNF, anti-proBDNF antibody or mBDNF before the extinction training day (Fig. 4*A*). As before, SD rats (SD) showed significantly higher freezing levels relative to control (CON) rats (effect of treatment: *F*_(4,30)_ = 113.53, *p* < 0.001; block 3–5: *p* < 0.001; Fig. 4*B*). Additionally, since the experimental procedures were different for the data shown in Figure 2 (three controls groups) and Figure 4, the freezing levels, which were tested 48 h after conditioning training, were qualified. However, no statistical difference was found between the SD groups of Figures 2, 4 (repeated-measures ANOVA, effect of treatment: *F*_(3,23)_ = 0.96, *p* > 0.05) or between CON groups of Figures 2, 4 (repeated-measures ANOVA, effect of treatment: *F*_(3,23)_ = 0.73, *p* > 0.05). vHPC infusion of proBDNF into SD rats was able to enhance fear extinction learning compared to SD (block 4: *post hoc p* < 0.01; block 5 and 6: *p* < 0.001; Fig. 4*B*). However, infusion of proBDNF antibody into control (CON-anti) rats were indistinguishable from CON rats (block 4–6: *p* < 0.001; Fig. 4*B*) and SD-proBDNF in their freezing levels (block 4–6: *p* < 0.001; Fig. 4*B*). Since previous studies found infusion of mBDNF into the IL mPFC promoted extinction memory of conditioned fear even in the absence of extinction training (Peters et al., 2010) and rescued the late-phase of long-term potentiation as well as the amnesia caused by protein synthesis inhibitors (Bekinschtein et al., 2008), firstly, in a parallel study, we have demonstrated that increased mBDNF exhibited the circadian oscillation in the vHPC (data not shown), which was consistent with the reports from other labs (Eckel-Mahan et al., 2008); and then intra-hippocampus injection of mBDNF was performed during SD (SD-mBDNF) to detect the effect of mBDNF on extinction acquisition. Although SD-mBDNF group learned extinction faster than SD group (block 5 and 6: *p* < 0.01; Fig. 4*B*), it was still slower than CON and SD-proBDNF groups (block 5 and 6: *p* < 0.01; Fig. 4*B*). However, infusion of cleavage-resistant proBDNF into the vHPC at ZT0 could not ameliorate the consolidation defects of SD rats (effect of treatment: *F*_(2,18)_ = 24.58, *p* < 0.001; *post hoc p* < 0.01; Fig. 4*B*). It suggested the impairment of consolidation process was not attributed to the disruptive effect of SD on circadian proBDNF expression. Together all, these results demonstrated that acquisition of extinction was associated with circadian proBDNF oscillation in the vHPC.

Growing evidences suggested extinction memory was linked with the activation of NMDA receptors (Burgos-Robles et al., 2007; Sotres-Bayon et al., 2009). For example, extinction of inhibitory avoidance learning was blocked with a variety of kinase, NMDA, mRNA, and protein synthesis inhibitors into the dorsal HPC (Vianna et al., 2001; Cammarota et al., 2003). Other studies have demonstrated that proBDNF directly or indirectly modiﬁed NMDA receptors. Early evidence implied the role of post-synaptically BDNF increased NMDA receptor activity through phosphorylation and upregulation of NMDA receptor subunits (Lin et al., 1998). Moreover, the proBDNF-p75^NTR^ signaling was important for the expression of NR2B-dependent LTD (Woo et al., 2005), which was required for the extinction of conditioned fear and taste aversion (Dalton et al., 2012; Li et al., 2016). More importantly, it has been suggested that the impairment in synaptic plasticity following SD may be due to a reduction in hippocampal levels of BDNF (Schmitt et al., 2016), alternation in NR2A/NR2B ratio of HPC NMDARs (Kopp et al., 2006) and without alternation receptor subunit composition (McDermott et al., 2006). Strikingly, this change was reversed after recovery sleep (Kopp et al., 2006). It was possible, therefore, that the disruption of circadian proBDNF oscillation in SD group, which showed extinction deficits, involved in NMDA receptors signaling. To test this hypothesis, fear-conditioned rats were sleep deprived 30 min preceded by intra-hippocampal infusion combinations of saline and saline (Sal-Sal), saline and proBDNF (Sal-proBDNF), CPP and proBDNF (CPP-proBDNF), NVP and proBDNF (NVP-proBDNF), or Ro25 and proBDNF (Ro25-proBDNF), and then assessed in extinction learning test as above (Fig. 4*A*). Both CPP (effect of treatment: *F*_(4,35)_ = 141.73, *p* < 0.001; block 4–6: *p* < 0.001; Fig. 4*C*) and Ro25 (block 4–6: *p* < 0.001; Fig. 4*C*) made acquisition of extinction learning more difficulty. However, NVP-proBDNF rats were indistinguishable from Sal-proBDNF rats in their freezing level (block 4: *p* < 0.01, block 5 and 6: *p* < 0.001; Fig. 4*C*), which demonstrated that proBDNF-NMDA NR2B, but not NR2A, signaling involved in SD-induced deficits in extinction acquisition.

To identify whether the proBDNF infusion could effectively reverse the circadian maximum of proBDNF levels, separated groups of rats were chosen and the circadian rhythmicity were detected as above time points. The expression of vHPC proBDNF varies with the circadian cycle in both CON and SD-proBDNF groups (effect of treatment: *F*_(3,20)_ = 57.91, *p* < 0.001; Fig. 4*D*). Both these groups had a higher proBDNF activity at ZT4 than SD group (both comparisons: *p* < 0.001), indicated intra-hippocampal infusion of proBDNF maintained the circadian pattern of proBDNF rhythm. However, although injection of mBDNF could slightly enhanced proBDNF level at ZT4 compared SD group (*p* < 0.05), this improvement still lagged behind pro-BDNF group as indicated by the significantly difference between SD-proBDNF and SD-mBDNF at ZT4 (*p* < 0.01). Additionally, we did not observe similar circadian pattern of proBDNF expression in CPP-proBDNF group (data not shown). Collectively these results indicated the infusion of proBDNF were sufficient for maintenance of its circadian rhythm, which could mitigate SD-induced fear extinction deficits.

### SD inactivated the proBDNF-mediated vHPC-IL, but not BLA-IL, projection to impair the acquisition of fear extinction

**Figure 5.**
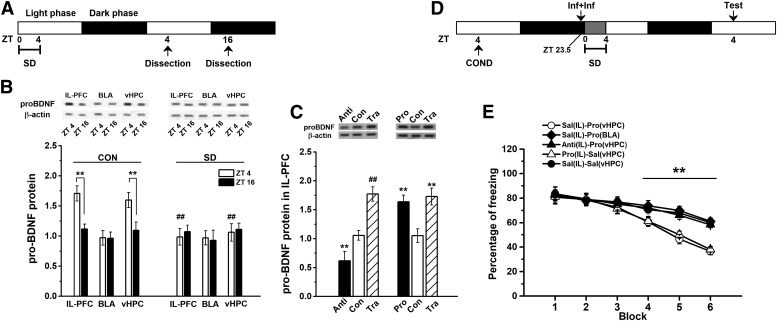
Circadian proBDNF expression in vHPC projections to the IL involved in SD-induced impairment of acquisition of extinction. ***A***, Treatment procedure of tissue collection. Rats were killed at ZT4 or ZT16 1 d after contextual conditioning, and IL-PFC, BLA, and vHPC were dissected. Note: the extracts that collected at ZT16 were conducted under red light. ***B***, The expression of proBDNF in IL, BLA, and vHPC under CON (control, left) and SD (right) condition; **p < 0.01 versus ZT16; ##*P* < 0.01 versus its matched tissue of CON condition; *n* = 6 per time point. ***C***, The expression of proBDNF in vHPC 0.5 h following the injection of anti-proBDNF antibody (Anti), cleavage-resistant proBDNF (Pro), or saline (Con), or 24 h following extinction learning (Tra); **p < 0.01 versus the matched Con group; ##*P* < 0.01 versus the matched Anti or Con groups; *n* = 7 per group. ***D***, Treatment procedure of extinction test. Following fear conditioning, rats received post-SD infusion(s) and then tested at the following ZT4. ***E***, The effect of co-infusion of saline into the IL and proBDNF into vHPC [Sal(IL)-Pro(vHPC)], saline into the IL and proBDNF into BLA [Sal(IL)-Pro(BLA)], anti-proBDNF antibody into the IL and proBDNF into vHPC [Anti(IL)-Pro(vHPC)], proBDNF into the IL and saline into vHPC [Pro(IL)-Sal(vHPC)], saline into the IL and saline into vHPC [Sal(IL)-Sal(vHPC)] on freezing level in SD rats; **p < 0.01: Sal(IL)-Pro(vHPC) or Pro(IL)-Sal(vHPC) versus Sal(IL)-Pro(BLA), Anti(IL)- Pro(vHPC) or Sal(IL)-Sal(vHPC); *n* = 7 per group.

The neural circuits, including vHPC, BLA, and IL-PFC, mediated extinction of conditioned fear (Peters et al., 2010; Sierra-Mercado et al., 2011; Rosas-Vidal et al., 2014). Since HPC and BLA inputs were important for supplying BDNF to the IL-PFC to facilitate extinction memory (Sierra-Mercado et al., 2011; Kimbrough and Biggs, 2014; Rosas-Vidal et al., 2014), we tested if the circadian rhythms of proBDNF activity were co-occurred in three brain regions throughout the circadian cycle (Fig. 5*A*). Intriguingly, the circadian oscillation was presented in both IL-PFC (compared ZT4 to ZT16: *p* < 0.01; Fig. 5*B*, left) and vHPC (compared ZT4 to ZT16: *p* < 0.01; Fig. 5*B*, left), but not BLA, while they disappeared when tested on day following SD (comparison between SD and CON in IL or HPC at ZT4: both *p* < 0.01; Fig. 5*B*, right). To detect whether the levels of proBDNF in the vHPC affect proBDNF levels in the IL-PFC, anti-proBDNF antibody (Anti) or cleavage-resistant proBDNF (Pro) was injected into the vHPC, and the proBDNF expression of the IL-PFC was assessed 0.5 h following the injection. The infusion anti-proBDNF antibody into vHPC significantly decreased the levels of proBDNF in the IL-PFC (effect of treatment: *F*_(2,18)_ = 86.92, *p* < 0.001; *post hoc*: *p* < 0.01; Fig. 5*C*, left), while the infusion of proBDNF significantly increased IL-PFC proBDNF levels (effect of treatment: *F*_(2,18)_ = 136.51, *p* < 0.001; *post hoc*: *p* < 0.01; Fig. 5*C*, right). Furthermore, the levels in the IL-PFC of the rats (Tra), which were subjected to extinction training, were detected 24 following training. The IL-PFC proBDNF was significantly enhanced compared with Anti and Con groups (both *p* < 0.01), but no statistical difference was found between Tra and Pro groups.

To seek whether maintaining of the co-occurred circadian rhythms was able to rescue the deteriorated effect of SD on extinction acquisition, rats were trained in fear conditioning and co-administration of saline in the IL-PFC and proBDNF in the HPC [Sal(IL)-Pro(HPC)], saline in the IL-PFC and proBDNF in the BLA [Sal(IL)-Pro(BLA)], anti-proBDNF antibody in the IL-PFC and proBDNF in HPC [Anti(IL)-Pro(HPC)], proBDNF in the IL-PFC and saline in the HPC [Pro(IL)-Sal(HPC)], and saline in the IL-PFC and saline in the HPC [Sal(IL)-Sal(HPC); Fig. 5*D*]. Similar to proBDNF effect on the vHPC [Sal(IL)-Pro(HPC)], infusion it into the IL-PFC reduced fear [Pro(IL)-Sal(HPC)], as indicated by no difference in freezing behavior (effect of treatment: *F*_(4,35)_ = 163.65, *p* < 0.001; Fig. 5*E*). The effect of vHPC proBDNF could be prevented by co-administration of proBDNF-inactivating antibody in the IL-PFC (Anti(IL)-Pro(HPC); block 4–6: *p* < 0.01; Fig. 5*E*). However, BLA infusion of proBDNF did not reverse the extinction damage, as measure by the significant difference between Sal(IL)-Pro(BLA) and Sal(IL)-Sal(HPC) groups (block 4–6: *p* < 0.01; Fig. 5*E*). Together all, these findings implied: (1) the levels of proBDNF in the vHPC affect proBDNF levels in the IL-PFC; (2) the expression of proBDNF in the IL-PFC, which was the primary site of action for hippocampal proBDNF, was critical for extinction acquisition.

### SD suppressed IL-PFC neural activity during extinction of conditioned fear

**Figure 6.**
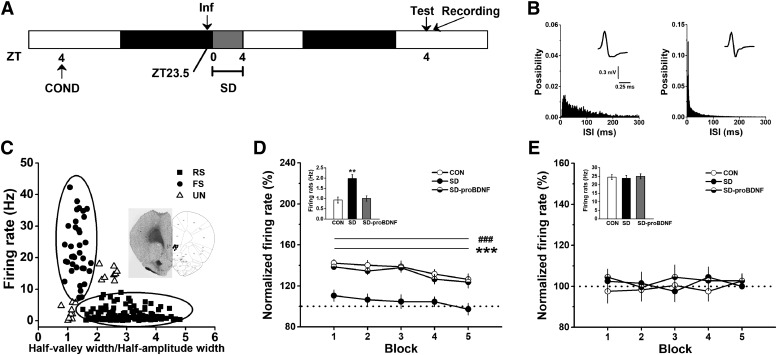
SD enhanced basal firing rate but inhibited response activity of IL-RS neurons. ***A***, Treatment procedure. Conditioned rats were infused proBDNF into vHPC 0.5 h before SD. Acquisition of extinction, which was performed at ZT4 on the next day, was followed by IL single-unit recoding. ***B***, Interspike interval (ISI) histograms of putative projection cell (left) and inhibitory interneuron (right). Insets were examples of average waveforms. ***C***, Distribution of average firing rate and half-valley to half-peak ratio of each IL neurons. Insets were the recording sites in the IL-PFC and the representative implantations of the electrode arrays. The ratio positively correlated with the spike width (Pearson’s *r* = 0.238; *p* < 0.001). Therefore, the wide spikes tended to have large half-valley to half-peak ratio. On the other side, a significant negative correlation between the mean firing rate and the ratio of half-valley to half-peak of the spike wave form was found (Pearson’s *r* = –0.371; *p* < 0.001). Thus, the high firing rate cells tended to have spike waveforms with relatively small half-valley to half-peak ratio. Cells were therefore separated into two groups as indicated by oval lines. Five-min courses of mean firing rate of projection neurons (***D***) and inhibitory interneurons (***E***); ***p < 0.001: CON versus SD; ###*p* < 0.001: SD-proBDNF versus SD. Insets were the spike frequency from baseline recording, which was conducted in the fear conditioning chamber 30 min before extinction test; **p < 0.01. Data were shown in 1-min blocks.

Our results clearly indicated that the circadian rhythm of proBDNF in vHPC could influence extinction via IL-PFC. Given the essential role of proBDNF in regulation of neural activity (Nagappan et al., 2009; Yang et al., 2014; Glerup et al., 2016) and the facilitation of IL-PFC excitability during extinction training (Santini et al., 2008; Milad and Quirk, 2012), in a separated group of fear-conditioned rats were sleep deprived preceded by proBDNF infusion or not, and the single-unit activity of IL-PFC was assessed following extinction learning (Fig. 6*A*). Neuron spike trains were classiﬁed by wave form shape (Fig. 6*B*) and spiking patterns (Fig. 6*C*). Three hundred sixteen single units were isolated from the IL of nine rats during three recording sessions. As shown in Figure 6*B*,*C*, wide-wave form neurons were classiﬁed as regular-firing neurons (87 from CON group, 87.9% of group population; 91 from SD group, 87.5% of the population; 97 from SD-proBDNF group, 85.8% of the population) while narrow-wave form neurons were classiﬁed as FS interneurons (12 from control group, 12.1% of the population; 13 from SD group, 12.5% of the population; 16 from SD-proBDNF group, 14.2% of the population). Additionally, 17 cells were not classified, since they could not be identified clearly (Fig. 6*C*). SD group showed a significant increase in basal firing rate of RS projection neurons compared to CON (*p* < 0.01) and SD-proBDNF (*p* < 0.01) group (Fig. 6*D*, inset). However, the basal firing rate of FS inhibitory interneurons was comparable among three groups (Fig. 6*E*, inset). Following fear extinction training, the normalized spiking frequency of RS neurons from SD group was diminished compared to those from CON group (effect of treatment: *F*_(2,272)_ = 268.19, *p* < 0.001; block 1–5: *p* < 0.001; Fig. 6*D*), while infusion of proBDNF resulted in an elevation of firing rate (block 1–5: *p* < 0.001; Fig. 6*D*), even turn back to the normal level. No main effect of treatment was found in FS interneurons (*F*_(2,38)_ = 1.53, *p* > 0.05; Fig. 6*E*). Additionally, Z-scores were also calculated and no statistical difference was found in putative projection cell or inhibitory interneuron (data not shown). These findings indicated that SD-induced circadian proBDNF rhythm disruption may impair fear extinction by suppressing post-training neural activity.

## Discussion

Endogenous processes referred to as circadian oscillators generate many of the daily rhythms in physiology and behavior of a variety of animals including humans. The present study provides multiple lines of evidence demonstrating that proBDNF activity in the vHPC maintained circadian oscillations, while SD between ZT0 and ZT4 severely lowered the enhancement during the day phase. We discovered SD suppressed acquisition of conditioned fear extinction was associated with the peak of proBDNF, which involved in the function of NR2B subunit of NMDA receptor, during circadian cycle. Furthermore, SD attenuated the projection of vHPC-IL as indicated by the synchronous absent circadian proBDNF in the IL-PFC and the abnormal facilitation of basal firing rate of RS neurons. Infusion of proBDNF to activate the circadian oscillations between vHPC and IL-PFC could effectively mitigate SD-induced deficits in acquisition of extinction and elevate sustained extinction-related activity of RS neurons in the IL-PFC.

Recently, growing researches have been renewed interest in circadian influence in learning and memory (Gerstner and Yin, 2010; Michel et al., 2013). Previous studies pointed on the circadian mBDNF expression and its related signaling were required for memory consolidation and stabilization (Eckel-Mahan et al., 2008; Gerstner and Yin, 2010; Coria-Lucero et al., 2016). Here we found vHPC proBDNF, the precursor of mBDNF, facilitated the extinction of conditioned fear memory, while ablation of proBDNF oscillations by SD, interfering the early stage of sleep or pharmacological blocking proBDNF activity during sleep, resulted in deficits in extinction of contextual fear memory. Since learning and memory function was based on biological processes and most biological processes were rhythmic, it should not surprising that circadian rhythms were seen in extinction of learned behaviors. However, proBDNF infusion can reverse the extinction defects even in the absent of early-stage sleep. Several studies showed that proBDNF preferentially activated p75^NTR^ to mediate neuronal cell apoptosis and neurogenesis (Je et al., 2012; Sun et al., 2012), which were required for early stage of sleep to selectively trim newly formed synapses in eliminating and maintaining of memory traces (Griffith and Rosbash, 2008; Vyazovskiy et al., 2008; Li et al., 2017). Compared with robustly effect of proBDNF, we also found that infusion of mBDNF tended to some extent rescue extinction defects. The effect from mBDNF itself or the feedback excitation of mBDNF on proBDNF could be the potential explanations, although the proximate mechanisms were not known. Therefore, in agreement with previous findings (Griffith and Rosbash, 2008; Yang et al., 2014), proBDNF most probably played a critical role in sleep homeostasis to remold neural circuitry.

Memory traces were typically thought to manifest as plastic changes in neuronal physiology. By activation of NR2B-containing NMDA receptors, proBDNF directly facilitated hippocampal LTD (Yang et al., 2014; Mizui et al., 2015), which served to eliminate fear memory through weakening the unused synaptic connection (Dalton et al., 2012; Li et al., 2016). Fear extinction depended on NMDARs activation (Peters et al., 2010; Dalton et al., 2012). Extendingly, we identified an obligatory role for NR2B-containing NMDA receptors in conveying the effect of circadian proBDNF on acquisition of extinction. It was concurred with previous findings that the function of NR2B-, but notNR2A-, containing NMDA receptors involved in the enhancement of acquisition of extinction memory (Corcoran et al., 2013; Otis et al., 2014). Furthermore, neural correlates of plasticity-related neurophysiology were also regulated by circadian rhythms (Chaudhury et al., 2005; Gerstner and Yin, 2010). Intriguingly, we also found that SD enhanced basal excitability in IL-RS neurons but not inhibitory FS interneurons, which was confirmed by that SD disrupted to establish the tight balance between excitation and inhibition (Griffith and Rosbash, 2008; Vyazovskiy et al., 2009). This excitation-inhibition balance may also contribute to hippocampal proBDNF/p75^NTR^ signaling during subjective night (Riffault et al., 2014). Indeed, the synaptic homeostasis hypothesis of sleep-wake regulation proposed a homeostatic increase in net synaptic strength and neural excitability along with decreased inducibility of associative synaptic potentiation due to saturation after SD (Kuhn et al., 2016). Given that proBDNF/p75^NTR^ mediated synaptic depression boosted network performance by protecting neural networks from saturation (Fino et al., 2005; He et al., 2015), the degradation of circadian proBDNF expression induced by SD most probably affected homeostatic changes in neuronal synchronization. Noteworthy, we demonstrated that the inhibited neural responses that occurred post-acquisition of conditioned extinction was not due to the enhanced neuronal excitability, since the normalized IL-RS frequency from SD was still lowered. In agreement with this observation, cortical mean firing rates changed as a function of sleep homeostasis, and this higher firing sustained in behavioral states (Vyazovskiy et al., 2009). Therefore, the circadian effect on neural excitability could be considered as naturally occurring form of metaplasticity (Finnie and Nader, 2012), in that the synaptic efficacy for an identical stimulation varied based on light/dark cycle (Chaudhury et al., 2005).

In contrast to the well-established function of HPC-BLA-mPFC circuit during contextual fear conditioning (Sotres-Bayon et al., 2009; Sierra-Mercado et al., 2011), we observed that infusing proBDNF into vHPC, but not BLA, attenuated learning extinction deficits in SD condition. Furthermore, blocking IL with anti-proBDNF antibody eliminated the proBDNF effect in the vHPC. Although there was no evidence that proBDNF was directly released from the vHPC inputs to IL-PFC, our findings clearly implied proBDNF infused into vHPC could affect the expression of proBDNF in the IL-PFC. The possible explanation is the infusion of proBDNF could be directly or indirectly transported into IL or the infusion of proBDNF could increase vHPC excitability leading to increased release of proBDNF in IL. Further evidence may come from transgenic animals and optogenetics. Several findings pointed out SD induced the aberrant HPC-PFC communication, as others have hypothesized that similar findings were a result of decreased consolidation of HPC-based memories during sleep (Mander et al., 2013) or improper HPC engagement during PFC-mediated tasks (Egan et al., 2003). For example, an increase in HPC activity and reduced long-term memory recall following sleep was observed with decreased synchronized activity between HPC and PFC (Mander et al., 2013). Other lab reported an exaggerated increase in low theta power in the HPC of Bdnf-e4 mice during freezing (Hill et al., 2016). This observation was consistent with HPC over-activation in individuals with the Val66Met polymorphism (Mascetti et al., 2013). Taken together, our results supported the vHPC-IL, but not BLA-IL, circuit engaged in proBDNF-mediated sleep homeostasis.

The combined rodent-human extinction findings predicted that extinguish fear responses and its associated network would be impaired in post-traumatic stress disorder (PTSD) patients (Milad et al., 2009; Sierra-Mercado et al., 2011). Consistent with mPFC dysfunction, PTSD patients showed normal conditioning and within-session extinction, but were unable to recall extinction memory (Milad et al., 2009). In healthy subjects, acquisition and retrieval of extinction was associated with increased activity in the vmPFC (homologous to rodent IL) and HPC (Milad et al., 2007). PTSD subjects exhibited deactivation in these areas, coupled with hyperactivation of the dorsal anterior cingulate cortex (homologous to rodent PL; Milad et al., 2009). Our findings suggested that it was during extinction training when activity in these areas was important. Sleep was maintained or disturbed might predict an individual’s ability to recover from a preexisting psychiatric condition (Pace-Schott et al., 2015; Kuhn et al., 2016; Schmitt et al., 2016). Restorative sleep was necessary to maintain adequate BDNF activity, which was notably reduced in patients with PTSD and was increased following psychiatric treatment (Felmingham et al., 2013; Zhang et al., 2014). Therefore, sleep was a key mediator at the connection between trauma exposure and the BDNF system (Andero and Ressler, 2012; Milad and Quirk, 2012), for which deregulation can exacerbate the mental and physical comorbidity (Andero and Ressler, 2012; Hill et al., 2016). Our study given the prospect that improved sleep quality may result in psychiatric recovery, which was associated with reactivity proBDNF signaling.

In conclusion, the proBDNF expression underwent circadian oscillations in HPC-IL pathway that correlated with the ability to acquisition of conditioned extinction. SD induced deficits in extinction learning, but not conditioning acquisition. The disruption of the circadian proBDNF levels induced by SD involved in NR2B, but not NR2A, subunits of NMDA receptors. Furthermore, SD enhanced the basal firing rate but depressed response activity of IL-RS neurons, which was reversed by providing proBDNF during early stage of sleep. Our findings suggested that proBDNF quite possible engaged in regulation of sleep homeostasis by balancing excitation and inhibition in the projection of HPC-IL. Further studies should focus on the role of proBDNF activity in extinguishing unwanted memory to offer potential therapeutic strategies for psychiatric disorders.

## Synthesis

Reviewing Editor: Cheryl Sisk, Michigan State University Decisions are customarily a result of the Reviewing Editor and the peer reviewers coming together and discussing their recommendations until a consensus is reached. When revisions are invited, a fact-based synthesis statement explaining their decision and outlining what is needed to prepare a revision will be listed below. The following reviewer(s) agreed to reveal their identity: Ingrid Liu, Junuk Lee.

Please address the following in your revised manuscript.

1. Results Section 3.3: Add “Figure 3A” (missing word); line 430

2. Figure 2: What was the purpose of testing Post-COND SD memory at 20 h? And why was this not also done for Post-COND SD (4-8) and Pre-COND?

3. Figure 4 depicts nice results used to support figure 2. The authors should consider putting the two figures together in order for the readers to better understand the relationship between the two.

4. Fig 6C: labels are needed for the Western blots.

5. Figure 7E y-axis: spelling error “Normalized”. In addition, please clarify whether z-scores were used in the firing rate analysis?

## Author Response

Responses to the comments of reviewers：

Thanks for the referee's comments and each question has been carefully answered as following. All the responses and changes are highlighted and marked in blue color in the revised version of manuscript.

Synthesis Statement for Author (Required):

Please address the following in your revised manuscript; these revisions require editorial review only.

1. Results Section 3.3: Add “Figure 3A” (missing word); line 430

Response: Many thanks for reminding us of this. We have added it in the revision.

2. Figure 2: What was the purpose of testing Post-COND SD memory at 20 h? And why was this not also done for Post-COND SD (4-8) and Pre-COND?

Response: Many thanks for reviewer's useful comments. We tested at 20 h (pre-extinction at ZT24) was to make sure that conditioned fear was successful acquired in sleep deprivation and control groups. Therefore, the significant difference in the extinction stage was not attributed to the differences in the acquisition of fear conditioning. We have also tested at 20 h (pre-extinction at ZT24) in the Post-COND SD (4-8) or Pre-COND experiments. Similarly, there was no statistical difference in the freeing levels between sleep deprivation and control groups. We have mentioned this in the revised manuscript.

3. Figure 4 depicts nice results used to support figure 2. The authors should consider putting the two figures together in order for the readers to better understand the relationship between the two.

Response: Many thanks for the helpful suggestion. We have put these two figures together in the revision.

4. Fig 6C: labels are needed for the Western blots.

Response: Many thanks for reminding us of this. We have added the labels in the revised manuscript.

5. Figure 7E y-axis: spelling error “Normalized”. In addition, please clarify whether z-scores were used in the firing rate analysis?

Response: Many thanks for reviewer's reminding. We have corrected it in the revision. Z-scores were also calculated and no statistical difference was found in putative projection cell or inhibitory interneuron. We have mentioned this in the section 3.5 of the revision.
